# Minimally invasive off pump CABG technique using "LIMA to LAD" first in staged hybrid approach for complex Coronary artery disease

**DOI:** 10.1186/1749-8090-10-S1-A119

**Published:** 2015-12-16

**Authors:** Lalit Mohan Joshi, Bhuwan Chandra Tewari, Sudarshan Kumar Vijay, Subhash Singh Rajput, Dhramendra Kumar Srivastava, Satyawati Deswal, Satish Chandra Dhasmana, Praveen Kumar Das

**Affiliations:** 1 Department of Cardiothoracic and Vascular Surgery, RMLIMS, Lucknow, India; 2 Department of Cardiology, RMLIMS, Lucknow, India; 3 Department of Nuclear Medicine, RMLIMS, Lucknow, India; 4 Department of Critical care and Anesthesiology, RMLIMS, Lucknow, India

## Background/Introduction

Best solution to solve ideal revascularisation remains a dilemma for complex Coronary artery disease. However, emerging minimally invasive off pump CABG technique can provided optimum solution for this condition.

## Aims/Objectives

To evaluate significance of Minimally invasive CABG using " LIMA to LAD first " in staged hybrid approach in patients with complex Coronary artery disease with compromised LV function and associated co morbid conditions.

## Method

Between December 2012 and 2014 total 28 patients were taken up for Surgery using off pump MIDCAB and MICS CABG technique. LIMA was harvested in all cases using harmonic scalpel and target LAD grafting was done using polypropylene 7-0 or 8 - 0 depending on luminal size and quality of LIMA. All patients were evaluated with preoperative risk stratification and standard work up protocol including consent to convert in sternotomy, on pump or any other life support system in demand.

## Results

Out of 28 patients, 27 (97%) had successful LIMA to LAD grafting achieved. All patients were operated off pump method.

Relief in angina was observed in 60 per sent cases. Nine patients (30 %) underwent staged PCI after six months of the CABG. They also had smooth course and improved outcome during and after the intervention.

## Discussion/Conclusion

Using current Minimally invasive off pump CABG technique with staged hybrid PCI option for non - LAD lesions, complex Coronary artery lesion can be efficiently managed by the heart team with better outcomes in short term. In addition,this procedure may provide safe and cohesive environment for the Surgeons and intervention cardiologists to work together for best interest of the patients.

